# Thyroid disorders and pregnancy

**DOI:** 10.1186/1756-6614-6-S2-A26

**Published:** 2013-04-05

**Authors:** Małgorzata Karbownik-Lewińska

**Affiliations:** 1 Department of Oncological Endocrinology, Medical University of Łódź, Łódź, Poland; 2 Department of Endocrinology and Metabolic Diseases, Polish Mother’s Memorial Hospital – Research Institute, Łódź, Poland

## 

During pregnancy specific changes in thyroid physiology occur, resulting in such consequences as different course of thyroid disorders and difficulties in diagnostics of thyroid disorders. Pregnancy is characterized by the increased formation of thyroid hormones. This is associated with the substantial increase in the requirements for dietary iodine – according to current recommendations to 250 micrograms/day. Therefore, additional iodine supplementation is advised at the level of 150 micrograms/day to be administered to every pregnant and lactating woman, and also to women who are planning to be pregnant. Another physiological change during pregnancy is thyroid hyperstimulation, caused by human chorionic gonadotrophin (hCG) in the first trimester. Quite frequently it takes the form of gestational transient thyrotoxicosis. Thyroid dysfunctions, i.e. hyper- and hypothyroidism, both are predominantly of autoimmune etiology in pregnant women. Thus, hyperthyroidism is usually associated with Graves’ disease, whereas hypothyroidism – with Hashimoto’s thyroiditis. The diagnosis is based on abnormal values of thyroid hormones and thyrotropin concentrations, with some difficulties in the interpretation of results occurring mainly in the first trimester. The increased concentration of thyroid antibodies can be helpful to diagnose thyroid dysfunction of autoimmune origin, therefore they should always be measured. Medical treatment in hyperthyroid pregnant women is the management of choice, with propylthiouracil being the preferred antithyroid drug in the first trimester and thiamazole being recommended in the second and third trimesters. Careful control of maternal thyroid function is required during antithyroid drug treatment to avoid fetal hypothyroidism. In turn, hypothyroidism, especially its subclinical form, relatively frequently occurs during preconception period and gestation. Replacement therapy with levothyroxine (L-T4) is the treatment of choice in hypothyroidism. Hypothyroid patients on L-T4 replacement should be carefully monitored to keep TSH and thyroid hormone concentrations in recommended ranges before conception and during pregnancy. Patients with pre-existing hypothyroidism generally require increased L-T4 doses during pregnancy. Discussing the diagnostic procedures used in pregnant women constitutes the separate issue. For example, not free thyroid hormones but their total fractions are recommended for the diagnosis of thyroid dysfunction during gestation. Another separate issue relates to screening for thyroid dysfunction before and during pregnancy. According to current guidelines, universal screening is recommended in individuals at high risk for thyroid illness. Summing up, the diagnosis and management of thyroid disorders during pregnancy differ substantially from those in general population.

